# Inhibitory effects of human lactoferrin on U14 cervical carcinoma through upregulation of the immune response

**DOI:** 10.3892/ol.2013.1776

**Published:** 2013-12-27

**Authors:** HUAIPING SHI, WENYE LI

**Affiliations:** College of Animal Science and Technology, Northwest A&F University, Yangling, Shaanxi 712100, P.R. China

**Keywords:** human lactoferrin, antitumor, immunomodulatory, U14 cell, cervical carcinoma

## Abstract

Human lactoferrin (hLF) is a multifunctional glycoprotein that inhibits cancer growth. However, the inhibitory effect of this glycoprotein in cervical cancer remains inconclusive. This study investigated the efficacy of hLF on the inhibition of U14 cervical cancer *in vivo*. Recombinant adenovirus carrying hLF (Ad-hLF) were constructed. Mice inoculated with U14 cells were randomly allocated to four treatments: i) Phosphate-buffered saline (negative control), ii) Ad-green fluorescent protein (negative control), iii) Ad-hLF (studied) or iv) cyclophosphamide (CTX; positive control). Tumor growth, as well as levels of natural killer (NK) cells, CD4^+^ and CD8^+^ peripheral blood T lymphocyte subpopulations, serum cytokines and vascular endothelial growth factor (VEGF) in tumor tissues were detected. Compared with the negative controls, tumor growth was inhibited by hLF and mice lifespans in the Ad-hLF-treated group were prolonged to reach the levels of the CTX-treated group. The activity of tumor-killing NK cells was upregulated by hLF. Moreover, the number of CD4^+^ and CD8^+^ peripheral blood T lymphocyte subpopulations increased following treatment with Ad-hLF. Treatment with Ad-hLF increased the levels of serum interferon-γ, serum interleukin-2 (IL-2) and tumor necrosis factor-α, and decreased the levels of serum IL-4 in tumor-bearing mice. The expression of VEGF in tumor tissues was downregulated by hLF. In conclusion, hLF inhibits the growth of U14 solid tumors by modulating the immune response of tumor-bearing mice.

## Introduction

Lactoferrin is an 80-kDa member of the transferrin family of iron-binding glycoproteins (1,2). This protein is produced by epithelial cells and is found in mucosal secretions, including tears, saliva, nasal exudates, gastrointestinal fluids, and seminal and vaginal fluids (3). It is an important component of the non-specific immune system, with antimicrobial properties against bacteria, fungi and several viruses (4–7). Numerous functions of lactoferrin relate to immune activation and modulation (8).

Recent evidence indicates that lactoferrin also possesses potent *in vivo* activity against cancer cells (9–11). Subcutaneous administration of lactoferrin demonstrated an inhibitory effect on both tumor-induced angiogenesis and tumor growth in mice. In addition, intratumoral injections of bovine lactoferrin slow the growth of fibrosarcoma cells subcutaneously (s.c.) injected in mice (10). Furthermore, oral administration of bovine lactoferrin to rats that had been previously injected with azoxymethane to promote colon carcinogenesis results in an 83% reduction in the incidence of colon adenocarcinomas (11). A similar effect was achieved by oral administration of bovine lactoferrin, which may protect against colon carcinogenesis, suggesting that lactoferrin may be an effective therapeutic agent for cancer treatment.

Although the available evidence favors a direct inhibitory effect of lactoferrin on cancer cell growth and metastasis, little is known regarding the mechanism by which lactoferrin exerts its anticancer activity. In this study, we focused on the effect of human lactoferrin (hLF) overexpression via adenoviral gene transfer on uterine cervical carcinoma *in vivo*. We demonstrated that adenovirus carrying hLF (Ad-hLF) significantly inhibited U14 solid tumor growth. Natural killer (NK) cell activity and the number of CD4^+^ and CD8^+^ T lymphocyte cells in the peripheral blood of tumor-bearing mice were increased by Ad-hLF. We also identified that the levels of interleukin-2 (IL-2), interferon-γ (IFN-γ) and tumor necrosis factor-α (TNF-α) were increased, and the IL-4 level was decreased by Ad-hLF. Finally, vascular endothelial growth factor (VEGF) expression in tumor tissues was downregulated by Ad-hLF. These results suggest that tumor inhibition induced by lactoferrin is the result of immunomodulation.

## Materials and methods

### Cell lines and animals

BJ5183 *E. coli* was purchased from Invitrogen (Shanghai, China). The human embryonic kidney (HEK)-293 cell line was purchased from Microbix Biosystem, Inc. (Toronto, ON, Canada). The cervical cancer U14 cell line was obtained from the Institute of Medical Material, Chinese Academy of Medical Sciences (Beijing, China). The YAC-1 cell line was obtained from the Institute of Biochemistry and Cell Biology (Shanghai, China). Female Kunming mice (6–8 weeks old, weighing 18–22 g) were provided by the Experimental Animal Center of Xiehe Medical University (Beijing, China). The experimental use of mice was approved by the animal ethics committee of Xiehe Medical University.

### Construction of recombinant adenoviral vectors

Construction of recombinant adenovirus was performed as described previously (12). Briefly, the internal ribosome entrance site (IRES) system was employed to express hLF and green fluorescent protein (GFP) from the same cytomegalovirus (CMV) promoter. pAd-hLF and pAd-GFP plasmid vectors were purified through the BJ5183 *E. coli* and then transfected into HEK-293 cells. Ad-hLF was purified by cesium chloride ultracentrifugation at 80,000 × g for 20 h. A recombinant adenovirus carrying the GFP protein under the control of the CMV promoter (Ad-GFP) was used as a control vector. hLF expression in U14 cells following Ad-hLF transfection was detected.

### Western blot analysis

Cell extracts were separated by electrophoresis on 10 SDS-polyacrylamide gels, transferred onto nitrocellulose papers and probed with a mouse anti-hLF antibody (Santa Cruz Biotechnology, Inc., Santa Cruz, CA, USA). The mouse anti-hLF antibody was detected with a polyclonal goat anti-mouse Ig coupled to horseradish peroxidase (HRP) followed by enhanced chemiluminescence, with SuperSignal ECL western blotting detection reagents (Pierce Chemical Co., Rockford, IL, USA).

### In vivo studies

Mice (six per group) were s.c. injected with 1×10^7^ cells/ml U14 cells into the left axilla and then monitored daily for tumor growth. When tumors grew to 0.2–0.3 cm^3^, the mice were intratumorally (i.t.) injected with Ad-hLF (1×10^9^ pfu) or Ad-GFP (1×10^9^ pfu). Groups administered intratumorally with 100 μl phosphate-buffered saline (PBS) and 25 mg/kg cyclophosphamide (CTX) once every other day, for a total of seven times were used as the negative and positive controls, respectively. Tumor volumes were calculated using the equation V (mm^3^) = ab^2^/2, where a is the largest diameter and b is the perpendicular diameter. On day 14, mice were sacrificed and tumor growth was determined.

### NK cell activity in response to Ad-hLF

Spleen cells were collected as effector cells. Additionally, YAC-1 cells were cultured as target cells. Subsequently, effector and YAC-1 cells were mixed according to the ratio of 50:1 and cultured together. There were two control groups: Natural release group, in which target cells were cultured with RPMI-1640 medium; and maximum release group, in which target cells were treated with 1% NP-40 solution. After the cells in each group were incubated at 37°C for 2 h, the supernatants were collected and transferred to another well to incubate for 10 min at 37°C. Then, lactate dehydrogenase (LDH) substrate solution was added and incubated for 10 min, followed by termination of the enzymatic reaction with HCl. Finally, optical density in each group was detected with am iMark 500-nm microplate reader (Bio-Rad, Hercules, CA, USA) for analyzing NK cell activity.

### Flow cytometry assay

To investigate the effect of Ad-hLF on peripheral blood T-lymphocyte subpopulations of tumor-bearing mice, the collected anticoagulated blood from the Ad-hLF-treated group was diluted to 1×10^6^ cells/ml, labeled with anti-mouse monoclonal antibodies (4A Biotech Co., Ltd., Beijing, China) for 30 min and then incubated for 30 min at 4°C with rabbit anti-mouse FITC-IgG monoclonal antibodies to CD4^+^ and CD8^+^ (4A Biotech Co., Ltd.). Stained cells were examined with an EPICS-XL FACSCalibur flow cytometer using EXPO 32 ADC software (Beckman Coulter, Fullerton, CA, USA).

### Enzyme-linked immunosorbent assay (ELISA)

Prior to sacrifice by cervical dislocation, the blood of tumor-bearing mice was collected, deposited for 1 h at 4°C and centrifuged for 15 min at 600 × g. Subsequently, the serum was collected and IL-4, IL-2, IFN-γ and TNF-α levels were determined using a commercial ELISA kit (Zhongshan Golden Bridge Biotechnology Co., Ltd., Beijing, China) according to the manufacturer’s instructions.

### VEGF analyses by immunohistochemistry

According to SP kit descriptions (Zhongshan Golden Bridge Biotechnology Co., Ltd.), the paraffin sections were deparaffinized and then dipped into gradient ethyl alcohol. After the antigens were fixed, the sections were incubated for 10 min with 3% H_2_O_2_ at room temperature and then the rabbit anti-VEGF antibody (Santa Cruz Biotechnology, Inc.) was added. Following incubation overnight at 4°C, a polyclonal goat anti-rabbit Ig coupled to HRP secondary antibody (Santa Cruz Biotechnology, Inc.) was added to the sections and incubated for 30 min at room temperature. Finally, the sections were dyed with diaminobenzidine (DAB) and hematoxylin, and then observed under an Olympus fluorescence microscope (Shanghai Lai Electronic Technology Co., Ltd., Shanghai, China). The nuclei of positive cells containing VEGF were dyed brown and the nuclei of negative cells were dyed blue. Twenty fields of view were randomly selected. The total number of cells was counted in each field to calculate the percentage of positive cells.

### Statistical analysis

Statistical analysis was performed using Student’s t-test. Different letter superscripts between values in the histograms indicate a significant difference (P<0.05) and same letter superscripts between values indicate no significant difference (P>0.05).

## Results

### hLF expression in U14 cells

**Figure 1:**
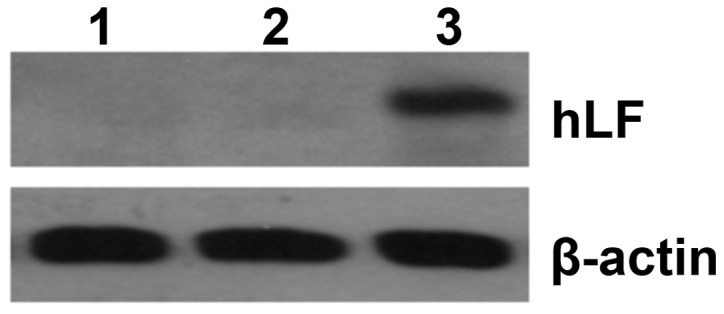
Expression of hLF in U14 cells. Following Ad-hLF transfection, U14 cells were lysed and hLF expression was detected by western blotting. Lane 1, non-treated U14 cells; lane 2, U14 cells treated with Ad-GFP; lane 3, U14 cells transfected with Ad-hLF. hLF, human lactoferrin; Ad-hLF, adenovirus carrying human lactoferrin; Ad-GFP, adenovirus carrying green fluorescent protein.

It was reported that the IRES sequence allows the expression of two different genes at high levels (13). We utilized the IRES system to express hLF and GFP from the same CMV promoter. The GFP and hLF genes were respectively cloned downstream and upstream of the IRES region under the control of a CMV promoter. After the recombinant adenoviral vector was constructed and transfected into HEK-293 cells, GFP proteins were clearly observed under a fluorescence microscope, suggesting that these vectors were normally transfected and proliferated in the HEK-293 cells. Subsequently, after a number of the recombinant adenoviral vectors were obtained from the HEK-293 cells, they were in turn transfected into the U14 cells, and western blotting revealed that the expression of hLF was significantly increased in U14 cells (Fig. 1), which greatly inhibited the growth of U14 cells.

### Effects of Ad-hLF on tumor growth in vivo

**Figure 2:**
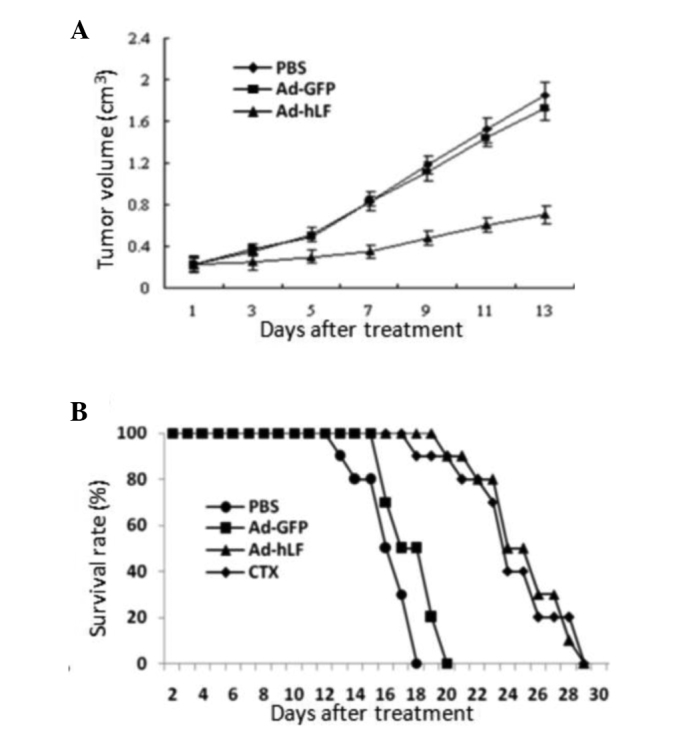
Effect of Ad-hLF on tumor growth and animal survival *in vivo*. Mice were subcutaneously injected with 1×10^7^ U14 cells/ml. When tumors grew to 0.2–0.3 cm^3^, mice were intratumorally injected with PBS, Ad-GFP (1×10^9^ pfu), Ad-hLF (1×10^9^ pfu) or CTX (25 mg/kg). (A) Tumor volume in each group was observed every other day. (B) Lifespan of mice in each group from day 2 was investigated every other day. Ad-hLF, adenovirus carrying human lactoferrin; Ad-GFP, adenovirus carrying green fluorescent protein; PBS, phosphate-buffered saline; CTX, cyclophosphamide.

To evaluate the *in vivo* antitumor activity of Ad-hLF, the U14 cells were i.t. injected with Ad-hLF (1×10^9^ pfu) or Ad-GFP (1×10^9^ pfu) into tumor-bearing mice, once every other day as described. Tumor growth was monitored daily. The PBS- and Ad-GFP-treated tumors kept growing uninterruptedly. The tumors in the Ad-hLF-treated group were markedly smaller compared with those of the Ad-GFP-treated group, when measured 14 days after seven injections (Fig. 2A). Moreover, survival of mice treated with Ad-hLF treatment was significantly prolonged compared with that of PBS- or Ad-GFP-treated mice (Fig. 2B). The lifespan of PBS- or Ad-GFP-treated mice was ~20 days, while the mice treated with Ad-hLF were still alive at the same time-point and their lifespans were prolonged to ~30 days, close to the CTX-treated group. These results indicate that Ad-hLF may suppress the growth of U14 solid tumors *in vivo*.

### NK cell activity in response to Ad-hLF

**Figure 3:**
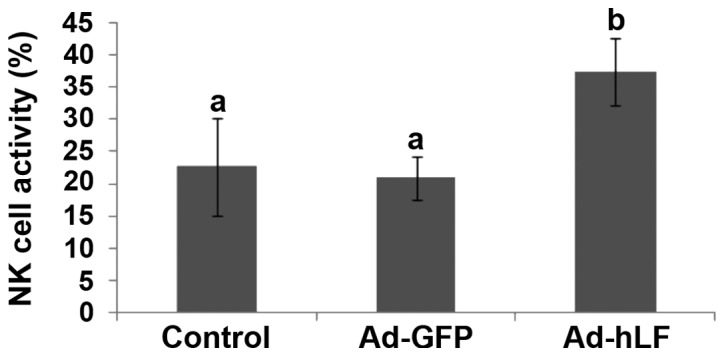
Spleen cells were the effector cells and YAC-1 cells were the target cells. Effector and target cells were mixed and cultured together. The two control groups were a natural release group and a maximum release group. After the cells were incubated, the supernatants were collected and then lactate dehydrogenase substrate solution was added. Finally, the OD was detected in each group with a 500-nm microplate reader. NK cell activity was determined through the following formula: X = (x−y)/(z−y), where X is NK cell activity, x is OD value of studied group, y is OD value of natural release group and z is OD value of maximum release group. NK cell activities in phosphate-buffered saline-, Ad-GFP- and Ad-hLF-treated groups were 22.53±7.62, 20.85±3.45 and 37.38±5.12%, respectively. Different letter superscripts between values indicate a significant difference (P<0.05) and same letter superscripts between values indicate no significant difference (P>0.05). OD, optical density; NK, natural killer; Ad-GFP, adenovirus carrying green fluorescent protein; Ad-hLF, adenovirus carrying human lactoferrin.

NK cells act as cytolytic effector lymphocytes. NK cells often lack antigen-specific cell surface receptors and are therefore involved in innate immunity, i.e. they are able to react immediately with no prior exposure to the pathogen (14). In mice and humans, NK cells play a role in tumor immunosurveillance by directly inducing tumor cell death (15,16). In the present study, we analyzed NK cell activity through the use of LDH. Fig. 3 shows that the percentage of NK cell activity in the Ad-hLF-treated group was significantly higher compared with that of the PBS- or Ad-GFP-treated control groups (P<0.05), which indicates that the activity of NK cells in killing the tumor is increased by Ad-hLF.

### Effect of Ad-hLF on peripheral blood T lymphocyte subpopulations

**Figure 4:**
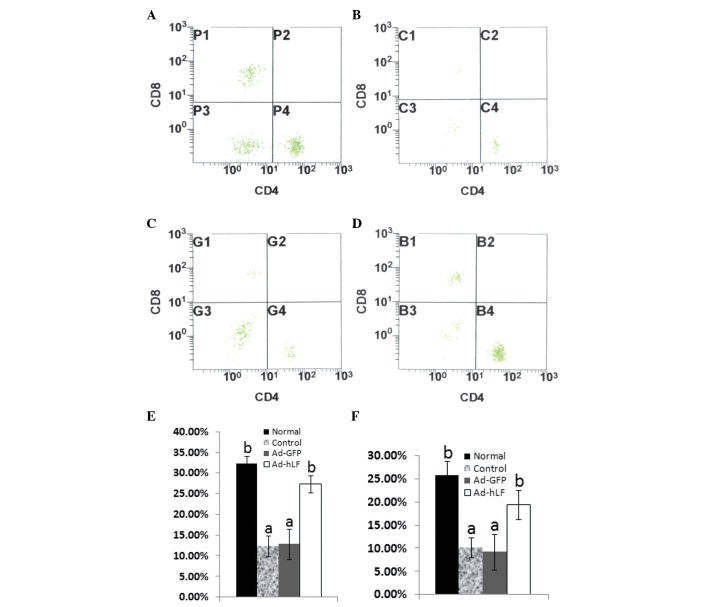
Collected anticoagulated blood from each group was diluted to 1×10^6^ cell/ml, labeled with anti-mouse monoclonal antibodies and then incubated for 30 min with rabbit anti-mouse FITC-IgG monoclonal antibodies to CD4^+^ and CD8^+^. Stained cells were examined with an EPICS-XL FACSCalibur flow cytometer using Expo 32 ADC software. (A) Normal mice; (B) PBS-treated mice; (C) Ad-GFP-treated mice; (D) Ad-hLF-treated mice. (E) The percentages of CD4^+^ T cells in PBS-, Ad-GFP- and Ad-hLF-treated mice and normal mice were 12.39±2.53, 12.85±3.62, 27.38±2.12 and 32.25±1.87%, respectively. (F) The percentages of CD8^+^ T cells in PBS-, Ad-GFP- and Ad-hLF-treated mice and normal mice were 10.24±2.17, 9.25±3.95, 19.52±3.14 and 25.79±3.14%, respectively. Different letter superscripts between values indicate a significant difference (P<0.05) and same letter superscripts between values indicate no significant difference (P>0.05). PBS, phosphate-buffered saline; Ad-GFP, adenovirus carrying green fluorescent protein; Ad-hLF, adenovirus carrying human lactoferrin.

The development and progression of tumors is greatly affected by the function of T lymphocytes (17). Mature T lymphocytes mainly differentiate into CD4^+^ and CD8^+^ cells. CD8^+^ cells activated by CD4^+^ cells destroy virally infected cells and tumor cells. The aforementioned two subpopulations in tumor-bearing mice were quantified by flow cytometry. The results demonstrated that the number of CD4^+^ and CD8^+^ T lymphocyte cells in the peripheral blood of tumor-bearing mice treated with Ad-hLF significantly increased compared with that of the control group (P<0.05) and was near to that of normal mice (P>0.05) (Fig. 4). These results demonstrate that Ad-hLF promotes the development of CD4^+^ and CD8^+^ cells in tumor-bearing mice.

### Effects of Ad-hLF on IL-2, IL-4, IFN-γ and TNF-α

**Figure 5:**
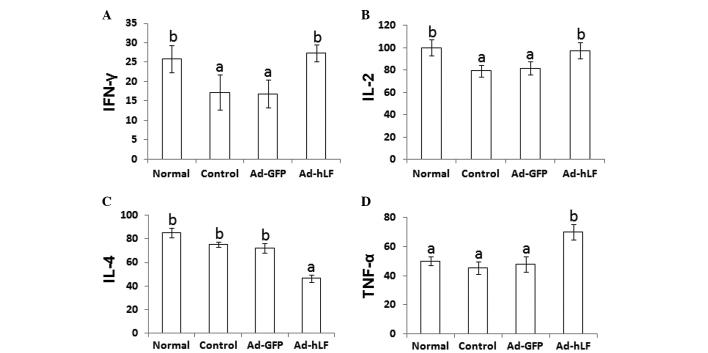
Blood from tumor-bearing mice was collected, deposited for 1 h at 4°C and centrifuged for 15 min. Subsequently, the serum was collected and IFN-γ, IL-2, IL-4 and TNF-α levels were determined using an enzyme-linked immunosorbent assay kit. (A) IFN-γ levels in phosphate-buffered saline (PBS)-, Ad-GFP- and Ad-hLF-treated mice and normal mice were 17.25±4.53, 16.85±3.62, 27.38±2.12 and 25.86±3.43 pg/ml, respectively. (B) IL-2 levels in PBS-, Ad-GFP- and Ad-hLF-treated mice and normal mice were 79.37±5.16, 81.75±6.15, 97.45±7.12, 100.13±7.27 pg/ml, respectively. (C) IL-4 levels in PBS-, Ad-GFP- and Ad-hLF-treated mice and normal mice were 75.24±2.17, 72.25±3.95, 46.52±3.14 and 85.13±4.18 pg/ml, respectively. (D) TNF-α levels in PBS-, Ad-GFP- and Ad-hLF-treated mice and normal mice were 45.32±4.35, 48.03±5.14, 70.12±5.15 and 50.21±3.12 pg/ml, respectively. Different letter superscripts between values indicate a significant difference (P<0.05) and same letter superscripts between values indicate no significant difference (P>0.05). IFN-γ, interferon-γ; IL, interleukin; TNF-α, tumor necrosis factor-α; Ad-GFP, adenovirus carrying green fluorescent protein; Ad-hLF, adenovirus carrying human lactoferrin.

After T helper (Th) cells are activated, they divide rapidly and secrete cytokines, including IL-2, IL-4, IFN-γ and TNF-α, which regulate or assist in the active immune response (18). To determine the effects of Ad-hLF on the secretion of cytokines, the serum levels of IL-2, IL-4, IFN-γ and TNF-α in tumor-bearing mice were analyzed by ELISA. When Ad-hLF was administered to the tumor-bearing mice, the levels of serum IFN-γ and IL-2 were significantly increased and were close to the levels in the normal group (P<0.05) (Fig. 5A and B). Compared with the other three groups, the level of serum IL-4 was significantly decreased, but the levels of serum TNF-α were markedly increased in the Ad-hLF-treated group (P<0.05) (Fig. 5C and D).

### VEGF expression in tumor tissue was decreased by Ad-hLF

**Figure 6:**
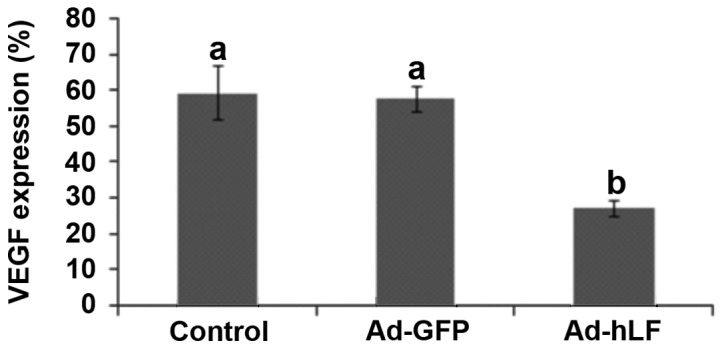
Expression of VEGF in tumor cells. After mice were treated with Ad-hLF, VEGF expression levels in tumor tissues were detected by immunohistochemistry. Subsequently, the number of VEGF-positive and -negative cells were counted to analyze VEGF expression. Expression rates of VEGF in PBS-, Ad-GFP- and Ad-hLF-treated groups were 59.39±7.53, 57.85±3.62 and 27.38±2.12%, respectively. Data are presented as the mean ± SD of three independent experiments. Different letter superscripts between values indicate a significant difference (P<0.05) and same letter superscripts between values indicate no significant difference (P>0.05). VEGF, vascular endothelial growth factor; Ad-hLF, adenovirus carrying human lactoferrin; Ad-GFP, adenovirus carrying green fluorescent protein; PBS, phosphate-buffered saline.

VEGF is a signal protein produced by cells that stimulates vasculogenesis and angiogenesis. When VEGF is overexpressed, it contributes to disease. Cancers that express VEGF are able to grow and metastasize (19). After mice were treated with Ad-hLF, we detected VEGF expression in tumor tissues using immunohistochemistry. Brown nuclei indicated VEGF-positive cells and blue nuclei indicated VEGF-negative cells. Our results demonstrated that numerous cell nuclei were brown, suggesting that VEGF was greatly expressed in the PBS- and Ad-GFP-treated control groups (data not shown). However, few cell nuclei were brown, but a number were blue in the Ad-hLF-treated group (data not shown), suggesting that VEGF was slightly expressed. Statistical analysis certified that the VEGF expression level in the Ad-hLF-treated group was lower compared with that of the other two groups (P<0.05) (Fig. 6). These results show that VEGF expression in tumor tissue is blocked by hLF.

## Discussion

Lactoferrin is becoming an increasingly important strategy for inhibiting carcinogenesis and tumor growth. Direct inhibition of cellular growth is one mechanism by which lactoferrin may inhibit the growth of numerous cancers. Lactoferrin treatment reduces colonic carcinogenesis in rats and decreases solid tumor growth and metastases in mice (11,20). hLF acts against the growth of solid tumors and the development of metastases in mice (21). Bovine lactoferrin inhibits lung metastasis of B16 melanoma and colon-26 tumor cells in mice and is protective against tongue, esophagus, intestinal, lung and bladder carcinogenesis in rats (9,22–25). The growth of cervical cancer is inhibited by Ad-hLF through the regulation of apoptotic factors (26). The present study extended the observation to cervical cancer and demonstrated the effects of Ad-hLF on the immune response of U14 cervical carcinoma-bearing mice. Our results indicated that the inhibitory roles of Ad-hLF on cervical cancer may be related to its upregulation of the immune response against tumors.

NK cells are a type of cytotoxic lymphocyte critical to the innate immune system. The role NK cells play is analogous to that of cytotoxic T cells in the vertebrate adaptive immune response (27). NK cells provide rapid responses to virally infected cells and respond to tumor formation, acting approximately three days after infection (28). After NK cell activity is promoted by hLF, NK cells release full granzymes to kill tumor cells. Small granules in the NK cell cytoplasm contain proteins, such as perforin and proteases known as granzymes. Upon release in close proximity to a cell marked for cell death, perforin forms pores in the cell membrane of the target cell, creating an aqueous channel through which the granzymes and associated molecules enter, inducing either apoptosis or osmotic cell lysis (29).

Cytokines play a crucial role in NK cell activation. Cytokines involved in NK activation include IL-12, IL-15, IL-18, IL-2 and CCL5 (29–31). Based on this, we investigated the level of serum IL-2 to determine if it was affected by hLF. In the present study, in which tumor-bearing mice were treated with Ad-hLF, the level of serum IL-2 was increased, which indicates that hLF activates NK cells. Once activated, NK cells work to control viral infections by secreting IFN-γ and TNF-α. IFN-γ activates macrophages for phagocytosis and lysis, and TNF-α promotes direct killing of tumor cells by NK cells (30,32). The increased levels of IFN-γ and TNF-α indicate that hLF is involved in NK cell activation. NK cells promote the expression of Fas on cancer cells. Fas is not normally expressed on tumor cells and therefore NK cells aided Fas-dependent apoptosis upon binding with Fas ligand (FasL)-expressing NK cells (33).

Aside from the inhibition of tumor cell growth by NK cells, T cells play an important role in tumor inhibition. Mature T lymphocytes mainly differentiate into CD4^+^ and CD8^+^ cells. CD4^+^ and CD8^+^ T cells strongly prevent tumor development (34,35). CD4^+^ T cells, which are a type of Th cell, induce the maturation of B cells into plasma cells and the activation of cytotoxic T cells, including CD8^+^ cells (36). CD8^+^ T cells destroy virally infected cells and tumor cells (37). We found that the number of CD4^+^ and CD8^+^ T cells in the peripheral blood of tumor-bearing mice treated with Ad-hLF significantly increased compared with that of the control group, which suggests that Ad-hLF increases the expression of CD4^+^ and CD8^+^ T cells and may activate them. Through the help of activated CD4^+^ T cells, CD8^+^ T cells are activated and migrate to the tumor site, producing a specific cytotoxic effect. Perforin and granzymes are released from CD8^+^ T cells. Perforin forms pores in the cell membrane of the target cell, creating an aqueous channel through which the granzymes enter, which leads to cell apoptosis via degradation of DNA of the target cell or stimulation of the FasL/Fas pathway.

Additionally, Th cells include two subsets with different functions: Th1 and Th2 cells. Once activated, these Th cells divide rapidly and secrete cytokines that regulate or assist in the active immune response. Th1 cells mainly secrete IL-2, IL-12, IFN-γ and TNF-α, which are involved in cellular immunity and graft rejection (38,39). Th2 cells mainly secrete IL-4, IL-5, IL-6 and IL-10, which are involved in humoral immune and allergic reactions (40). IL-2 or IFN-γ may induce NK cell-induced tumor death and IFN-γ directly causes DNA fragmentation of tumor cells, which demonstrates that Th1 cells present a powerful status against tumors (41,42). However, IL-4 promotes IL-10 in tumor tissue and the latter interferes with the expression of inflammatory factors, including IL-12 and IFN-γ, blocks the activation of NK cells and reduces the antigen formation of tumor cells in order to escape cytoimmunity against tumor growth (43–45). Therefore, a Thl and Th2 cell balance maintains a normal state of the body by secreting cytokines. If these cytokines in Thl and Th2 cells are dysregulated, the cytoimmunity is destroyed. In the present study, the levels of IL-2 and IFN-γ were significantly increased, but the levels of IL-4 were reduced by hLF in tumor-bearing mice. This caused the Th1/Th2 balance to favor Th1 progression, promoting the antitumor response. It was also demonstrated that TNF-α levels were increased by hLF and increased the antitumor effect. The aforementioned results demonstrated that hLF may inhibit the growth of cervical cancer by rescuing the balance between Th1 and Th2 cells and strongly activating Th1 cells in tumor-bearing mice.

Although we determined that cervical cancer was inhibited through the cytoimmunity initiated by hLF, it remains unclear how to directly inhibit the tumor by hLF. Due to the inhibitory role of IFN-γ with regard to tumor vascular formation, the expression of VEGF in tumor tissue was investigated (46). VEGF is a signal protein produced by cells and it stimulates vasculogenesis and angiogenesis (47). When VEGF is overexpressed, it contributes to disease (48). Solid cancers, including cervical cancer, do not grow beyond a limited size without an adequate blood supply. Once released, VEGF may elicit several responses. It may cause a cell to survive, move or further differentiate (49). After Ad-hLF was i.t. injected, VEGF expression in tumor tissue was significantly decreased, which suggests that Ad-hLF may directly downregulate VEGF so that the vascular components in the tumor are not formed, causing inhibition of cervical cancer.

In conclusion, the immune response of tumor-bearing mice was upregulated by hLF. hLF increased the activity of NK cells and rescued the balance of serum cytokines in tumor tissue. Furthermore, hLF upregulated the function of T cells and blocked the expression of VEGF. Due to the initiation of cytoimmunity against tumor cells by hLF, cervical cancer was suppressed.
